# Measuring climate change’s impact on different sugarcane varieties production in the South of Goiás

**DOI:** 10.1038/s41598-023-36582-7

**Published:** 2023-07-19

**Authors:** Thiago Vizine Da Cruz, Ricardo Luiz Machado

**Affiliations:** Pontifical Catholic University of Goiás, Pontifical Catholic University of Goiás. Avenida Universitária, nº 1440, Área 3, Bloco D, 3ºandar, Setor Universitário, 74065-010 Goiânia, Goiás, Brasil

**Keywords:** Climate change, Climate-change impacts

## Abstract

A crucial aspect analysed during the last years, aiming to improve sugarcane production, is the impact of climate change on sugarcane productivity. One of the strategies to mitigate climate change's impact on sugarcane yield is the development of new varieties known to positively affect crop production. This paper analysed how climate change impacts sugarcane production regarding the different planted varieties. Data regarding sugarcane harvest were collected from a cooperative in the south of Goiás state—Brazil, the second biggest national sugarcane producer. Results indicate that climate impact on sugarcane yield is irrelevant when controlling for different varieties. Considering the results presented in this work, the Brazilian government should keep the incentives for the development of new sugarcane varieties and, at the same time, spur sugarcane producers to use the new sugarcane varieties. The results imply that if the variety is correctly chosen, sugarcane can be produced without harming the environment, contributing to reaching SDG 15. Moreover, it is less probable that an adverse climatic event will destroy the planted area, preventing sugarcane producers from severe loss and contributing to achieving SDGs number 1 and 2.

## Introduction

Brazil is the largest sugarcane producer in the world, having produced more than 654 million tons in the 2020/2021 harvest. In the same year, Goiás produced 74.04 million tons of sugarcane^1^. These data place the State of Goiás as the second major Brazilian sugarcane producer, behind the State of São Paulo.

Brazil has a long history of developing new sugarcane varieties, having invested in this research for over 30 years^2^. Oliveira, Barbosa, and Daros^2^ state that in 2020, genetically modified sugarcane varieties provided solely by RIDESA (the oldest Brazilian sugarcane genetic research company) represented more than 60% of Brazilian sugarcane fields, having reached 68% in 2015^2^. The Brazilian Agricultural Research Company – EMBRAPA’s Technological Information Agency (EMBRAPA-AGEITEC)^3^ argues that a significant part of the rise in sugarcane productivity is related to using new sugarcane varieties.

According to some authors, the sugarcane production expansion results from occupying the natural area through deforestation, causing environmental and social issues^4–6^. Antonelli^7^, for example, argues that food production contributes heavily to climate change and pollution.

The former discussed issues denote the importance of research for increasing sustainable sugarcane production and reducing social and environmental impacts. An important step is to understand each variable's behaviour in sugarcane production. In this sense, some studies have tried to estimate the impact of specific variables on sugarcane production^8–12^.

One aspect that has been recently analysed is the impact of climate change on sugarcane productivity. Gbetibouo and Hassan^13^, Adhikari, Nejadhashemi and Woznicki^14^, Ali et al.^15^, and Knox et al.^16^, for example, agree that climate change has exerted and will employ a significant impact on agriculture, including sugarcane production. Climate change has sometimes led to severe economic and non-economic loss^17^. Wheeler and Von Braun^18^ state that climate change can endanger food production, slowing the progress toward the end of hunger and worsening food insecurity.

One approach to mitigate climate change's impact on sugarcane production and productivity is the development of new varieties^19–24^.

Despite its possible positive effects, developing new sugarcane varieties demand further crop-variety-specific research^25^. Nevertheless, few researchers, such as Linnenluecke et al.^26^ and Verma et al.^27^, researched this subject. Others, such as Dias and Sentelhas^23^, performed their analysis considering specific sugarcane varieties. This lack of information regarding sugarcane crops with different varieties indicates a considerable research gap once the varieties’ specificities may impact the final production.

The main objective of this paper is to analyse how climate change impacts sugarcane yield, considering the climatic variables and the different planted varieties. The hypothesis guiding this research argues that the climatic impacts on sugarcane productivity lose strength by controlling for sugarcane varieties.

The main research objective is aligned with United Nations Sustainable Development Goals (SDG) 2 (zero hunger) and 15 (life on land). Nevertheless, Aguilar-Rivera^28^ states that improving sugarcane production and productivity also collaborates to reach SDG number 1 (ending poverty in all its forms everywhere), 3 (ensuring healthy lives and promoting well-being for all at all ages), 7 (ensuring access to affordable, reliable, sustainable, and modern energy for all), 9 (building resilient infrastructure, promoting inclusive and sustainable industrialization and foster innovation).

## Climatic change and Goiás’ sugarcane production

### Sugarcane and climate variation

A critical approach for increasing sugarcane productivity is to understand each variable’s behaviour, enabling the structure of more reliable production models.

Ahmad et al.^29^ and Abdoulaye et al.^30^ highlight the importance of analysing the impact of climate change on sugarcane production. Singh et al.^31^ and Swami, Dave, and Parthasarathy^32^ argue that climate change has become a significant threat to sustainable agriculture. Santos et al.^19^ argue that climate change promoted greater crop demand, including higher water scarcity resistance and soil conditions difficulties. Khan, Shah, and Iftikhar-Ul-Husnain^33^ assert that climate change has led to global warming, unpredicted storms, drought, flood, and crop losses, affecting agricultural productivity and causing food insecurity. Akbar and Gheewala^34^ and Mulinde et al.^35^ discuss the possible threats climate change poses to agriculture and food production.

Khan, Shah, and Iftikhar-Ul-Husnain^33^, Silva et al.^36^, McGree et al.^37^, Kelkar, Kulkarni, and Rao^38^ and Chandio et al.^39^ analysed the impact of climate change on sugarcane production in different places and came to a similar conclusion. The authors claim that the amount of rain is positively related to the increase in productivity, while the increase in temperature has an inverse relationship. Singh et al.^31^ and Swami, Dave, and Parthasarathy^32^ agree that rising temperatures harm sugarcane yield. In its turn, Jyoti and Singh^40^ came to a different conclusion on the rainfall´s impact on sugarcane production. They argued that climate change and rainfall negatively affect Indian sugarcane yield.

Analysing the impact of climate change on the southeast region of Punjab, Pakistan, Akbar and Gheewala^34^ concluded that the evapotranspiration rate is expected to increase in the future, leading to an expansion in sugarcane and cotton crop water requirement. Linnenluecke et al.^26^ investigated how climate change impacted sugarcane output in different regions of Australia. The authors state that increased atmospheric carbon concentration and maximum temperatures have harmed sugarcane production after 1995. Linnenluecke et al.^26^ considered the impact of the different sugarcane varieties on sugarcane production. As far as we know, this is the only research considering this specificity in the econometric model.

Ahmad et al.^29^ concluded that irrigation, rainfall, and soil condition positively and significantly impact sugarcane production decrease in Pakistan. Heureux et al.^41^ studied how climate trends will impact Pakistan’s agricultural production in Indus River Basin. The authors concluded that temperatures rising caused by climate change would negatively affect the primary crop production in the region, including sugarcane production. Rehman et al.^42^ analysed how Pakistan’s agricultural crop is related to CO_2_ emission and discovered that sugarcane is positively associated with this gas, which has responsibility for global warming. Ncoyini, Savage, and Strydom^43^ state that climate change and the lack of access to climate change information by small-scale farmers have decreased sugarcane production in South Africa.

Some researchers however found no impact of extreme climate change on sugarcane production. Kumar et al.^44^ for example argues that neither Indian summer monsoon rainfall, nor the El Niño influenced in Indian sugarcane production during the analysed years. The authors claim that strategies adopted by sugarcane producers, such as irrigation were able to diminish climate impact influence. Others such as Ray et al.^45^ argue that climate change impact on crop production (including sugarcane) varies according to the region studied. Therefore, meanwhile climate change has reduced sugarcane production, in places such as Northern Africa, Sub Saharan Africa and Western, Southern and South-eastern Asia) in other regions climate change has increased sugarcane production (the Americas, Central and Eastern Asia and Western and Southern Europe).

Some authors tried to predict how climate change will impact sugarcane production in the future. Santos et al.^19^ simulated several scenarios through DSSAT/CANEGRO model for the production based at Alagoas, Brazil. The authors concluded that temperature rise will cause energy sugarcane yield to increase until 2060. Da Silva et al.^46^ warn that due to the lack of rainfall caused by climate change, in the future, a significant part of the sugarcane crop in the state of São Paulo will require irrigation. Jaiswal et al.^47^ used the CANEGRO-Sugarcane crop model to predict the influence of climate change on sugarcane production in the mid and long future. The authors conclude that a rise in the minimum and maximum temperature in the future will lead to an accelerated plant growth, but a shorter amount of sucrose.

Verma et al.^27^ used DSSAT-CSM-CANEGRO for simulating climate change impact on different Indian sugarcane varieties the authors demonstrate how climate change impact the different sugarcane varieties distinctively. Despite important, crop simulation must be analysed carefully. Yin et al.^48^ demonstrate that Chinese sugarcane crop simulation made from 1980 to 2009 presents wrong results, due to problems with the main models used.

### Sugarcane varieties

Sugarcane’s characteristics related to resisting adverse climatic situation, including high or low temperatures and drought issues, has already been observed by several authors^19–24,49^.

Different crop varieties require different crop-variety-specific research^25,50^. According to EMBRAPA-AGEITEC^3^, the sugarcane genetic improvement promoted by different laboratories aiming to better respond to soil and climate adversities and diseases has been paramount for increased sugarcane productivity in the past years. Antonelli^7^ states that genomic sequencing and gene-editing techniques are paramount to increasing crop production without harming the environment, resisting climate change, and resistance to pests and diseases. Ali et al.^15^ state that in order to mitigate the effects of climate change, it is necessary to develop different crop varieties.

Oliveira, Barbosa, and Daros^2^ state that the main target of sugarcane genetic improvement programs is productivity upgrading. IAC^51^ states that new varieties are being developed to guarantee better productivity, focusing on regional specificities. EMBRAPA-AGEITEC^3^ and CTC^52^ assert that several varieties must be used to ensure better results. Oliveira, Barbosa, and Daros^2^ present the main varieties used in Brazil in the 2019/2020 harvest. This information is replicated in Appendix A.

According to EMBRAPA-AGEITEC^3^, the main sugarcane varieties used in Brazil come from the following laboratories: Interuniversity Network for the Sugar Energy Sector Development (RIDESA), COPERSUCAR, Sugarcane Technology Center (CTC), and Campinas Agronomic Institute (IAC). Each sugarcane variety possesses different characteristics, making it adequate for different regions. EMBRAPA-AGEITEC^3^ displays the differences between the main sugarcane varieties used in Brazil. Table 1 summarizes the critical information.

**Table 1:** Critical differences between the main sugarcane varieties used in Brazil.

*Soil requirement*
Very demanding
SP77-5181, SP87-396, SP87-344, SP83-5073, RB85-5546
Demanding
RB85-5453, RB85-5036, SP80-1816, SP80-1842, SO87-365, SP80-3280, RB85-5536, SP86-155, SP79-1011, SP81-320, SP-911049
Little demanding
RB85-5156, RB83-5053, RB83-5486, RB84-5210, RB85-5113, SP86-42
Do not require
RB72-454, RB92-8064, RB83-5089, RB86-7515, RB86-5230, SP83-2847, RB85-5035, SP85-5077
*Maturation*
Super early
RB85-5156, SP87-396
Early
RB83-5054, RB85-5453, SP77-5181, RB85-5035, RB83-5486, SP83-5073, SP80-1842, SP86-155, IAC86-2210
Medium
SP81-3250, SP80-1816, RB84-5210, RB85-5536, SP87-365, RB86-5230, RB85-5113, RB92-8064, SP85-3877, SP86-42, SP83-2847
Late
RB72-454, RB83-5089, RB86-7515
*Transport yield*
Worse
RB83-5486, SP80-1842, RB83-5089, RB83-5054, RB85-5156
Regular
RB84-5210, SP80-1816
Good
SP79-1011, SP77-5181, RB72-454, RB85-5113, RB85-5536, RB84-5257, RB85-5453, SP79-2233, RB86-7515, RB92-8064, SP81-3250
*Mechanical harvesting*
Worst
RB83-5054, RB85-5156, RB83-5089
Bad
B83-5486
Good
SP79-1011, RB85-5453, SP80-3280, SP80-1816, SP81-3250, RB85-5113, RB72-454, SP-2233, RB86-7515, RB92-8064
*Herbicide sensitivity*
Very sensitive
RB85-5036, RB85-5113, SP87-365, RB86-5230, SP85-3877
Sensitive
RB83-5089, RB84-5210, SP80-1816, SP80-1842
*Flowering*
Every year
RB85-5035, RB85-5156, RB85-5453, RB84-5197, RB86-5230, SP83-2847
Regularly
SP80-1842, SP80-3280, RB83-5486, SP81-3250, SP87-365
Scarce
RB83-5089, RB80-6043, RB72-454, SP80-1816, RB86-7515, SP85-3877
Do not bloom
RB83-5054, RB85-5113, RB85-5536, RB84-5210, RB92-8064, SP79-1011, SP83-5073
*Drought tolerance*
RB86-7515, RB75-8540, SP79-1011, RB83-5054, SP80-1842, RB85-5002, RB85-5156, SP83-5073
Water-demanding
SP79-2233, RB85-5453, RB80-1816, RB85-5536, SP87-344, SP85-3877

*Source:* EMBRAPA-AGEITEC ^3^.

The information displayed in Table 1 indicate that it is essential to choose the suitable variety according to the region and the different characteristics of each farm.

Considering the significant disparity between different varieties, it becomes evident that sugarcane research must consider which variety is being studied to avoid spurious results.

### Sugarcane genetic research in Brazil

Brazil has a long history of genetic research related to sugarcane. As stated by Oliveira, Barbosa, and Daros^2^, in 1971, the Sugar and Alcohol Institute (IAA), which belonged to the Industry and Trade Ministry, created the Sugarcane Improvement National Program (Planalsucar), which main target was to increase the national sugarcane yield. According to Oliveira, Barbosa, and Daros^2^, the Planalsucar developed and launched the RB sugarcane variety, which would become one of the main sugarcane varieties used in Brazil. Today, the RB variety is developed by RIDESA, once the Planalsucar was closed in 1990. Gazaffi et al.^53^ state that in the 2015/2016 season, 68% of the sugarcane-grown area was composed of RB varieties. Machado Jr and Braga Jr^54^ argue that the RB varieties are among those with more longevity, and that even though some of the varieties were developed at the beginning of the 1990s, they are still among the best available options.

Various sugarcane genetic research has been developed in Brazil. As stated in the previous section, Brazil has four prominent sugarcane genetic research institutes: RIDESA, COPERSUCAR, CTC, and IAC. Rossetto et al.^55^ state that eight new sugarcane varieties are launched each year in Brazil, and that to increase sugarcane yield, producers must correctly choose the sugarcane variety that best suits their location.

Boschiero et al.^56^ agree that choosing the correct sugarcane variety is crucial for achieving better results.

Aiming to improve Brazilian sugarcane productivity, Dias et al.^57^ investigated the impact of silicon treatment in four different sugarcane varieties used in São Paulo: RB867515, RB72454, SP81-3250, and SP83-2847. The authors claim that the RB72454 and SP83-2847 varieties were more responsive (presented better productivity) than the others. Chapola et al.^58^ show that some sugarcane varieties responded better to the Orange Rust disease in Brazil. The authors conclude that over the years, sugarcane producers migrated to sugarcane varieties more resistant to the disease. After many years of genetic research, in 2017, the first genetically modified sugarcane cultivar (CTC20BT) was commercialized. Cheavegatti-Gianotto et al.^59^ present this variety´s development, deregulation, and commercialization.

## Research methodology

### Database

Data regarding sugarcane harvest were collected in a sugarcane cooperative in the south of Goiás. The data sent by the cooperative refers to the total area production, production separated by sugarcane variety, cuts separated by crop, the yield of each cultivar, and the city where the crop was harvested. All data refer to 2021 and are farm-level detailed. According to the sugarcane cooperative, 27 sugarcane varieties are used by farmers in that region. Table 2 presents the used sugarcane varieties, the frequency in the dataset, their percentual share of the production, and the cumulative percentage.

**Table 2:** Sugarcane varieties used by farms in the selected region at the South of Goiás.

Number	Variety	Frequency	Percentage	Cumulative percentage
1	CTC4	140	18.54	18.54
2	RB867515	101	13.38	31.92
3	RB966928	90	11.92	43.84
4	RB855453	88	11.66	55.5
5	IACSP95-5000	79	10.46	65.96
6	RB855156	57	7.55	73.51
7	IAC91-1099	39	5.17	78.68
8	CTC9003	38	5.03	83.71
9	RB965902	22	2.91	86.62
10	SP80-1816	17	2.25	88.87
11	IACSP95-5094	16	2.12	90.99
12	RB985476	15	1.99	92.98
13	RB855536	9	1.19	94.17
14	RB92579	9	1.19	95.36
15	CTC2	8	1.06	96.42
16	SP81-3250	5	0.66	97.08
17	CTC9001	3	0.4	97.48
18	RB928064	3	0.4	97.88
19	RB935744	3	0.4	98.28
20	SP80-3280	3	0.4	98.68
21	CTC6	2	0.26	98.94
22	CTC9002	2	0.26	99.2
23	SP83-2847	2	0.26	99.46
24	CV0618	1	0.13	99.59
25	IAC93-3046	1	0.13	99.72
26	RB835054	1	0.13	99.85
27	RB975952	1	0.13	99.98

Despite the vast number of varieties, the first eleven represent over 90% of the total production. Thus, aiming to avoid collinearity problems, only those eleven first varieties were considered in the analysis.

All climate data were collected according to the city where the farm is located. Data concerning climate, such as precipitation and average temperature, were collected at INMET^60^. CO_2_ emission data was collected at SEEG^61^ and concerned the year 2019, the last year with available data. According to Silva et al.^36^ results, the mean temperature is expected to present a negative relationship, while precipitation presents a positive relationship to sugarcane yield. Marengo et al.^62^ confirm that temperature variation impacts sugarcane yield. Concerning CO_2_ emissions, according to Linnenluecke et al.^26^, it is expected that this variable presents a positive impact on sugarcane yield.

Crop cost data and fixed costs were collected at CONAB^63^. The detailed production cost data are presented in Table 3.

**Table 3:** Crop and fixed costs separated by farm size.

Familiar agriculture farm		Company farm	
Crop cost	Fixed cost	Crop cost	Fixed cost
Machinery rent	Depreciation	Airplane cost	Depreciation
Employees	Maintenance	Own machinery costs	Maintenance
Rural management	Social charges	Employees	Insurance
Seeds	Insurance	Management	
Fertilization		Fertilization	
Pesticides		Pesticides	

It is essential to notice that the Brazilian cost structure differs from other countries, like Pakistan, where, according to Muzammil, Zahid, and Breuer^64^, a considerable share of sugarcane crop cost constitutes the irrigation structure. Moreover, besides adding cost to the production, the irrigation process may also increase pollution once it is based on diesel power. Following Ali, Zubair, and Hussain^65^, Crop Cost is expected to present a positive relationship with Sugarcane Yield.

In order to analyse the statistical characteristics (mean, standard deviation, maximum and minimum values) of the considered data, the descriptive statistics of the analysed variables was performed. Results are presented in Appendix B.

The analysis indicates that although Precipitation and Mean Temperature present slight variations across the region, the same cannot be said regarding CO_2_ emissions. Crop Cost also presents huge variation; however, it must be considered that crop area varies across the farmers, impacting crop cost.

### Methodology

The research methodology adopted in this work was based on the investigation performed by Silva et al.^36^. The authors gathered municipality-level data and performed a panel data analysis, controlling for the region in which data had been collected in the Paraíba State, Brazil. Despite the importance of the research made by the authors, in trying to perform a similar analysis in the state of Goiás, we considered it necessary to add some information to the model. This research performed a cross-section analysis due to the availability of data. Also, this work focused on a specific region in the south of the state of Goiás. Therefore, the soil interference in sugarcane production was neutralized.

Moreover, unlike Silva et al.^36^, this work used the sugarcane yield as a dependent variable. Ahmad et al.^29^ explain that climate change can severely impact sugarcane yield. Therefore, using the sugarcane yield of each sugarcane variable will provide a better understanding of the relationship between climate change and sugarcane production.

Following Ali, Zubair, and Hussain^65^, the total cost of fertilizer and machinery was used as the control variable. However, different from Ali, Zubair, and Hussain^65^, this work used each farm´s total crop expenditure and total fixed cost. As exposed before, those numbers are provided by CONAB and represent an average estimation of the farm’s expenditure, separated by the size of the farm. CONAB classifies the farms into Familiar Agriculture or Company, based on Brazilian law 11.326/2006 ^66^, which provides unique benefits to small farmers. Therefore, before collecting data from CONAB, all the farmers listed by the sugarcane cooperative were separated according to their size.

Linnenluecke et al.^26^, Chandio et al.^39^, and Rehman et al.^42^ argue that CO_2_ emissions can also impact sugarcane production. This work followed these authors and included the CO_2_ variable in the model.

After collecting the data, a Cluster Analysis was performed. The dendrogram was performed after Ward’s Linkage clustering, with Euclidean Dissimilarity measure, based on the average sugarcane yield of each variety of the 2021 harvest.

Afterward performing the Cluster analysis, data were prepared for the regression examination. For the regression analysis, the Stata 17 software was used. First, heteroskedasticity and multicollinearity tests were performed. Heteroskedasticity problems were solved using cluster standard errors. The variance inflation factor (VIF) test was used for the multicollinearity. Multicollinearity problems appeared after inserting Radiation and Humidity variables. In order to prevent the study from spurious results, following Gujarati and Porter^67^, those variables were retrieved from the study.

After the control tests, the regression analysis was performed using the model presented in the next section.

### Model

The econometric model used in the regression analysis is presented in Eq. (1):

1 $$\begin{aligned} \Upsilon i = & \beta 0 + \beta 1\;Precipitation + \beta 2\;Mean\; Temperature + \beta 3CO_{2} \\ & + \beta 4CUTi + \beta 15\;Crop \;cost + \beta 16 \;Fixed\; cost + u \\ \end{aligned}$$

Υi is the sugarcane yield of the analysed variety in the period. $\beta 0$ is the coefficient of the constant of the equation. Precipitation, Mean Temperature, and CO_2_ refer to the climatic information of each city where the farm is located. β4 is the number of times the crop has been cut (harvested). Crop cost and Fixed cost refers to the production cost. Finally, µ is the error term of the equation.

After the establishment of the econometric model, the regression analysis was performed after the Ordinary Least Squares method.

## Results

**Figure 1:**
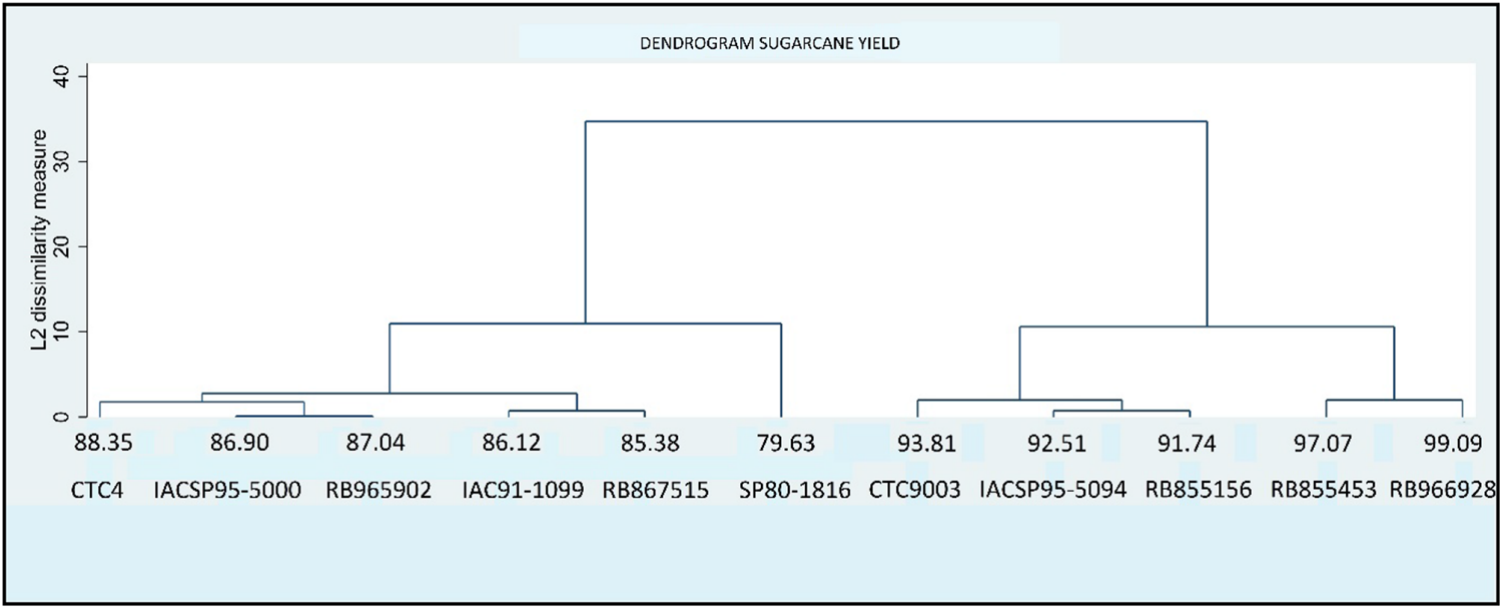
Dendrogram of analysed sugarcane varieties.

Figure 1 exhibits the dendrogram.

The Fig. 1 displays the average sugarcane yield of each variety and the name of each variety right below. As can be seen, there are four major groups. The first one comprises the CTC4, IACSP95-5000, RB965902, IAC91-1099, and RB867515 varieties. The second group is constituted solely by the SP80-1816 variety. The CTC9003, IACSP95-5094, and RB855156 varieties form the third one. The fourth group contains the RB855453 and the RB966928 varieties. Figure 1 shows that the SP80-1816 variety is less productive among the group.

Regarding production, the two most productive varieties (RB855453 and RB966928) represent only 1.10% of the total production (considering only the eleven most essential varieties production). The second most productive group – group number 3 – is responsible for 0.7% of the production, while group 2 (the less productive) answers for 0.06% of the total production. Most cooperative sugarcane production comprises group 1, with more than 98% of the total production.

Regarding the regression analysis, Table 4 presents the results:

**Table 4:** Climate change impact on sugarcane yield.

	(1)	(2)	(3)	(4)	(5)	(6)	(7)	(8)	(9)	(10)	(11)
Variables	Yield SP801816	Yield RB867515	Yield IACSP955000	Yield IACSP955094	Yield RB855156	Yield RB966928	Yield CTC4	Yield IAC911099	Yield RB855453	Yield RB965902	Yield CTC9003
Precipitation	0.106 (0.166)	− 0.109 (0.118)	− 0.0352 (0.0555)	− 0.0299 (0.0341)	− 0.0249 (0.0154)	0.0596 (0.134)	− 0.126 (0.125)	0.00435 (0.0503)	0.106* (0.0545)	− 0.00870 (0.0241)	0.0186 (0.0838)
Mean Temperature	11.34 (22.70)	− 4.393 (8.656)	0.754 (2.883)	0.00199 (1.374)	1.163 (0.846)	7.866 (5.526)	− 8.498 (9.558)	0.398 (1.553)	2.434 (2.827)	1.136 (1.034)	2.408 (3.806)
CO_2_ Emission	1.74e − 06 (1.77e − 06)	1.64e − 05 (1.03e − 05)	− 1.29e − 06 (6.73e − 06)	1.26e − 06 (3.99e − 06)	2.56e − 06 (1.78e − 06)	1.66e − 05 (1.15e − 05)	− 3.07e − 06 (1.13e − 05)	− 5.75e − 07 (4.18e − 06)	9.78e − 07 (5.65e − 06)	− 4.46e − 07 (2.20e − 06)	5.73e − 06 (1.10e − 05)
CutSP801816	14.74 (10.09)										
CutRB867515		13.57*** (2.135)									
CutIACSP955000			13.97*** (2.106)								
CutIACSP955094				27.71*** (6.432)							
CutRB855156					26.04*** (2.904)						
CutRB966928						34.00*** (8.213)					
CutCTC4							15.08*** (3.213)				
CutIAC911099								22.57*** (2.671)			
CutRB855453									13.73*** (1.866)		
CutRB965902										27.82*** (6.418)	
CutCTC9003											29.10*** (5.741)
Crop cost	5.24e − 06 (5.55e − 06)	− 4.73e − 06 (3.27e − 06)	9.06e − 07 (2.34e − 06)	− 2.45e − 07 (4.29e − 07)	− 2.64e − 06 (1.98e − 06)	− 1.65e − 06 (6.58e − 06)	4.65e − 06 (3.66e − 06)	− 7.82e − 08 (2.22e − 06)	6.52e − 06 (5.26e − 06)	− 8.66e − 06* (4.67e − 06)	− 3.49e − 07 (1.91e − 06)
Fixed cost	− 8.54e − 05 (0.000230)	− 0.000315 (0.000383)	0.000110 (0.000199)	− 0.000280 (0.000198)	0.000156 (0.000106)	0.000467 (0.000531)	− 0.000218 (0.000427)	6.82e − 05 (0.000108)	− 0.000221 (0.000355)	0.000295* (0.000156)	− 1.83e − 05 (0.000221)
Constant	− 410.5 (782.4)	287.8 (355.9)	21.70 (158.0)	75.24 (83.19)	− 13.73 (30.00)	− 338.5 (366.9)	416.2 (386.1)	− 21.39 (102.1)	− 171.1 (131.7)	− 42.22 (56.10)	− 84.51 (206.4)
Observations	41	41	41	41	41	41	41	41	41	41	41
R-squared	0.646	0.600	0.781	0.762	0.919	0.578	0.557	0.874	0.676	0.870	0.756

Standard errors in parentheses.

***p < 0.01, **p < 0.05, *p < 0.1.

Table 4 results indicate that neither rainfall, mean temperature, nor even CO_2_ emissions impact sugarcane yield for most of the varieties. Table 4 rainfall’s result is in line with^26,50^. Table 4 results show that climatic variables lose their importance in sugarcane yield when using the appropriate sugarcane variety.

Regarding the variable Precipitation, RB855453 (column 9) was the only sugarcane variety with significant result.

Finally, although small, sugarcane variety RB965902 (column 10) presented significant results for Crop Cost and Fixed Cost.

### Robustness check

**Figure 2:**
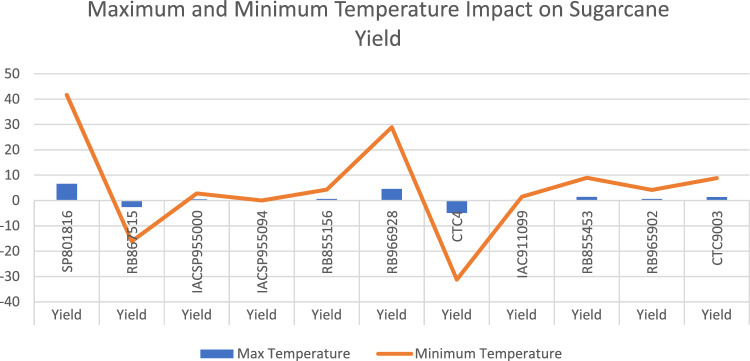
Maximum and minimum temperature impact on sugarcane yield.

The regression analysis was performed again to analyse how sugarcane production answered extreme climate conditions, replacing the mean temperature variable for maximum and minimum temperature. Due to collinearity problems, the regression was run separately. Figure 2 presents how Maximum and Minimum temperature impacts on the sugarcane yield of each variety. The tables containing the regression results can be found at Appendix C.

None of the presented results were significant. Nevertheless, it is possible to observe that Minimum temperature possess a larger impact on sugarcane yield that Maximum temperature.

### Robustness check 2–bootstrap analysis

Because of the small number of observations, a Bootstrap analysis was performed. The Bootstrap method was first presented by Efron and Tibshirani^68^ and, according to Joshi, Seidel-Morgenstern and Kremling^69^, it uses stochastic elements and repeated simulations to analyse the properties under consideration and overcome the theoretical limitations of a finite sample. The Bootstrap regression was performed considering the three temperature variables. The regressions are presented in Appendix D.

## Discussion

Precipitation results presented few significant results, indicating that precipitation does not affect sugarcane yield. In fact, in some cases, precipitation presented negative results (RB855156, column 5 Table C1 and Table D2, RB867515, column 2 Table D1 and Table D3). Those results confirm Ali et al.^15^, Knox et al.^16^, and Kumar et al.^44^. Regarding the Brazilian studies, we believe that the contradictory results presented by this research can be explained by two main reasons: 1° Silva et al. ^36^ conducted their research at Paraíba state, which is much drier than the South of Goiás. 2° Santos et al.^19^ and Da Silva et al.^46^ performed simulations for future crops, which provided forecasts, and not real interaction among the variables.

The lack of significant results in all temperature (mean, minimum and maximum) regressions confirms Knox et al.^16^ and Kumar et al.^44^ papers. However, even though it was insignificant, Fig. 2 and Tables C1, C2, D2 and D3 demonstrate that minimum temperature has a more decisive influence on sugarcane yield. This result aligns with Swami, Dave, and Parthasarathy^32^, who observed the same pattern in their study.

Moreover, Fig. 2 and Tables C2 and D3 results do not confirm Marengo et al.^62^. The authors demonstrate that several crop productions, including sugarcane, were impacted by the unusual cold conditions reported for the 2021 winter period (June–August). One possible explanation for the different results presented in this research is that most of the sugarcane production of Goiás state comes from the “Year Sugar” method, in which the sugarcane crop is planted between October and December and harvested after one year. Therefore, the cold event registered in 2021 did not affect the sugarcane crop of the South Region of Goiás because when the cold happened, the sugarcane crop was being harvested or on the verge of it.

Concerning the CO_2_ variable, the RB867515 variety (column 2) presented significant positive results for the CO_2_ Emissions in Table D1, and it was confirmed in Tables D2 and D3. This result means that, although small, the increase in temperature resulting from the increase in CO_2_ emissions may benefit this variety yield.

Relating to the CO_2_ emissions in Table 4, the lack of significance, contrasting with Linnenluecke et al.^26^ results, may be explained by the difference in the location of the sugarcane crop. As the state of Goiás is significantly closer to the equatorial line than Australia, the CO_2_ emissions in that region may provide a warmer climate, which benefits the sugarcane crop. It must be stressed that apart from significant results presented by the RB867515 variety in the Bootstrap regression (Tables D1, D2 and D3), all the other sugarcane varieties in all the other analyses (Tables 4, C1, C2, D1, D2 and D3) presented non-significant results, which confirms the hypothesis above.

Regarding the fact that our research confirmed so few results, we believe there are four possible explanations:

1. More control variables were inserted, guaranteeing that climatic variables presented their real impact without “aggregating” missing variables’ results. The Cut variable, for example, that represents how many times the crop has been harvested, which is critical information when analysing the sugarcane crop and presented significant results in almost all cases, is hardly ever used as a control variable. Silva et al.^36^, for example, only presented control variables for the different state regions. McGree et al.^37^ only used climatic variables in their model. The same problem can be observed in the research of Kelkar Kulkarni and Rao^38^. Ahmad et al.^29^ investigated separate climatic events, such as rainfall, without inserting temperature variables in their study.
2. As we controlled for the different sugarcane varieties, the climate variables’ impact lost strength; therefore, their results were not significant. Despite many authors’ awareness of the importance of accounting for different sugarcane varieties, only few authors such as Dias & Sentelhas^23^, Linnenluecke et al.^26^ and Verma et al.^27^ took this information under consideration.
3. The lack of information may have impacted the results presented (from this and other works). Knox et al.^16^ argue that the lack of data has led to contradictory and non-significant results in their work. The difficulty in having access to real data is an obstacle when researching the impacts of climate change on sugarcane yield. This situation may also explain the amount of work focusing on future forecasts, which may or may not provide correct answers once they cannot access all the future outcomes that may influence sugarcane yield. Indeed, it is possible to notice that many simulation research presents contradictory results when compared to those which had access to real data – Santos et al.^19^ and Silva et al.^36^ for Brazil, Singh et al.^31^ and Swami, Dave, and Parthasarathy^32^ for Maharashtra – India, for example. According to Yin et al.^48^, many crop simulations provided for China present wrong results due to problems with the models used, which underestimate multi-year sugarcane yield and fall short of simulating the pattern of interannual variability.
4. Results may change according to the region studied. Knox et al.^16^, Swami, Dave, and Parthasarathy^32^, Jyoti and Singh^40^, and Ray et al.^45^ proved it in their work. Moreover, more subtle facts can influence the climate change impact on sugarcane yield. Ncoyini, Savage, and Strydom^43^, for example, demonstrate that the deprivation of access to climate change information and the absence of know-how to deal with it increases the potential damage caused by climate change in South Africa.

## Conclusions

Since the beginning of the 1970s, Brazil has invested in the sugarcane genetic research. However, despite all the efforts made in the genetic field, only a small number of research have been made aiming to analyse how those new varieties interact with climate change conditions. This paper intended to answer this question.

Results indicate that climate conditions do not affect the sugarcane yield when controlling for the sugarcane variety. This conclusion does not align with most research regarding the relationship between sugarcane-climate change. However, as exposed in the previous chapters, most of the research does not consider the different sugarcane varieties. Moreover, most of them lack consistent control variables in their econometric model.

Results are essential to increase sugarcane productivity and its final production. It becomes clear that when controlling for the correct sugarcane varieties, the adverse climate conditions are minimized, making it possible to produce sugarcane without harming the environment, contributing to reaching SDG 15. Moreover, it is less probable that an adverse climatic event will destroy the planted area, preventing sugarcane producers from losing their investment. This result is essential if we consider the small producer, who has no condition to afford significant losses. This outcome is vital to achieving SDGs numbers 1 and 2.

Moreover, following Pienkowski et al.^70^ results, by helping to increase sugarcane production and diminish food insecurity, this work also contributes to SDG 3. According to the above-quoted authors, food insecurity may develop depression and mental health problems among the population.

Despite all the benefits provided by different sugarcane varieties regarding the climatic change, it is essential to notice that most of the varieties used by sugarcane producers of the studied region were developed in the 1990s. Machado Jr and Braga Jr^54^ claim that most Brazilian sugarcane production relies on “older” varieties. Therefore, considering the results presented in this work, in order to increase sustainable sugarcane production, preventing from endanger natural resources, restrain hunger, and without being threatened by climatic change, the Brazilian government should keep the incentives and sustain the development of new sugarcane varieties, and, at the same time, spur sugarcane producers to use the new sugarcane varieties.

Sugarcane genetic research is well-developed in Brazil, and several institutions promote different sugarcane varieties to surmount different production difficulties. However, this only happens for some sugarcane country producers. Hence, other countries' policymakers should elaborate long-term policies supporting the research and adoption of different sugarcane varieties. With the appropriate sugarcane variety, climate change impacts on sugarcane production will be mitigated, assuring there will not be a shortfall of production in the future.

Despite the originality of this work, it contains some limitations. First, it considered one-year data observation only. It is suggested that other studies with larger datasets are performed. Secondly, it observed only one production region. Different regions should be analyzed in future research to enhance the understanding of climate change impacts. Finally, the utilization of annual data does not provide a good overview of the relationship between climate change-sugarcane yield, once more critical than the amount of precipitation in the determined region when such rain falls. The same can be said regarding hot weather. When it comes to precipitation, for example, it must be analysed if it is raining when sugarcane is planted or not. Therefore, future research should use a monthly database indicating when sugarcane is planted and harvested.

## Supplementary Information

Supplementary Information.

## Data Availability

The data that support the findings of this study are available from the studied sugarcane cooperative but restrictions apply to the availability of these data, which were used under license for the current study, and so are not publicly available. Data are however available from the corresponding author upon reasonable request and with permission of the sugarcane cooperative.
