# Supplementary material for: Measuring climate change’s impact on different sugarcane varieties production in the South of Goiás
Source: Sci Rep. 2023 Jul 19;13:11637. doi: 10.1038/s41598-023-36582-7 (PMC10356802; doi:10.1038/s41598-023-36582-7)
Supplement: Supplementary file 1 — Supplementary Information. [file 41598_2023_36582_MOESM1_ESM.docx]

**Measuring Climate Change’s Impact on Different Sugarcane Varieties Production in the South of Goiás**

Thiago Vizine da Cruz

Ricardo Luiz Machado

# APPENDIX A

Table A1 - Main Sugarcane Varieties Planted in Brazil in 2019/-2020 Harvest

| **Classification** | **Variety** | **Total (ha)** | **%** |
| --- | --- | --- | --- |
| 1 | RB867515 | 1,133,512 | 21.4 |
| 2 | RB966928 | 676,693 | 12.8 |
| 3 | RB92579 | 487,022 | 9.2 |
| 4 | CTC4 | 446,538 | 8.4 |
| 5 | RB855156 | 223,187 | 4.2 |
| 6 | RB855453 | 178,168 | 3.4 |
| 7 | SP83-2847 | 128,980 | 2.4 |
| 8 | CTC9001 | 97,151 | 1.8 |
| 9 | CTC15 | 91,775 | 1.7 |
| 10 | RB855536 | 86,099 | 1.6 |
| 11 | SP80-1816 | 72,753 | 1.4 |
| 12 | IAC95-5000 | 72,472 | 1.4 |
| 13 | SP83-5073 | 72,442 | 1.4 |
| 14 | SP81-3250 | 69,770 | 1.3 |
| 15 | IAC91-1099 | 69,041 | 1.3 |
| 16 | SP80-3280 | 68,418 | 1.3 |
| 17 | CTC2 | 65,586 | 1.2 |
| 18 | SP91-1049 | 62,321 | 1.2 |
| 19 | CTC9003 | 54,990 | 1.0 |
| 20 | RB835054 | 50,204 | 0.9 |
| 21 | CTC20 | 49,462 | 0.9 |
| 22 | CV7870 | 48,589 | 0.9 |
| 23 | RB975201 | 44,691 | 0.8 |
| 24 | RB965902 | 42,942 | 0.8 |
| 25 | SP79-1011 | 42,238 | 0.8 |
| 26 | SP80-1842 | 40,076 | 0.8 |
| 27 | RB928064 | 38,979 | 0.7 |
| 28 | SP78-4764 | 35,103 | 0.7 |
| 29 | CTC9002 | 32,594 | 0.6 |
| 30 | CV6654 | 27,910 | 0.5 |
| Others | | 676,934 | 12.8 |
| Total | | 5,286,619 | |

Source: Oliveira, Barbosa, and Daros [2]

# APPENDIX b

Table B1 – Descriptive Statistics

| **Variable** | **Unit** | **Observations** | **Mean** | **Standard deviation** | **Min** | **Max** |
| --- | --- | --- | --- | --- | --- | --- |
| YieldSP801816 | Tons per hectare | 41 | 9.10 | 28.70 | 0.00 | 119.32 |
| YieldRB867515 | Tons per hectare | 41 | 38.77 | 46.28 | 0.00 | 130.00 |
| YieldIACSP955000 | Tons per hectare | 41 | 32.45 | 42.20 | 0.00 | 109.49 |
| YieldIACSP955094 | Tons per hectare | 41 | 10.11 | 27.98 | 0.00 | 100.00 |
| YieldRB855156 | Tons per hectare | 41 | 16.91 | 35.40 | 0.00 | 109.92 |
| YieldEB966928 | Tons per hectare | 41 | 43.33 | 61.26 | 0.00 | 288.03 |
| YieldCTC4 | Tons per hectare | 41 | 41.37 | 48.36 | 0.00 | 130.00 |
| YieldIAC911099 | Tons per hectare | 41 | 19.24 | 37.36 | 0.00 | 117.03 |
| YieldRB855453 | Tons per hectare | 41 | 32.50 | 46.70 | 0.00 | 120.00 |
| YieldRB965902 | Tons per hectare | 41 | 10.61 | 29.90 | 0.00 | 131.36 |
| YieldCTC9003 | Tons per hectare | 41 | 16.00 | 36.32 | 0.00 | 120.00 |
| Precipitation | Mm | 41 | 1,332.69 | 83.67 | 1,267.00 | 1,439.2. |
| Mean Temperature | ºC | 41 | 24.04 | 0.91 | 19.65 | 24.6. |
| Max Temperature | ºC | 41 | 40.01 | 1.13 | 33.10 | 40.5. |
| Min Temperature | ºC | 41 | 8.06 | 1.43 | 6.20 | 9.2. |
| CO^2^ Emission | Tons of CO_2_ /year | 41 | 1,233,199 | 655,973.80 | 101,614.00 | 2,475,223 |
| cutSP801816 | Year | 41 | 0.41 | 1.61 | 0.00 | 9.00 |
| cutRB867515 | Year | 41 | 1.97 | 2.80 | 0.00 | 9.00 |
| cutIACSP955000 | Year | 41 | 1.87 | 2.56 | 0.00 | 8.00 |
| cutIACSP955094 | Year | 41 | 0.29 | 0.87 | 0.00 | 4.00 |
| cutRB855156 | Year | 41 | 0.65 | 1.38 | 0.00 | 5.00 |
| cutRB966928 | Year | 41 | 1.09 | 1.46 | 0.00 | 5.00 |
| cutCTC4 | Year | 41 | 1.39 | 2.06 | 0.00 | 8.00 |
| cutIAC911099 | Year | 41 | 0.78 | 1.52 | 0.00 | 5.00 |
| cutRB855453 | Year | 41 | 1.53 | 2.50 | 0.00 | 8.00 |
| cutRB965902 | Year | 41 | 0.48 | 1.38 | 0.00 | 6.00 |
| cutCTC9003 | Year | 41 | 0.46 | 1.12 | 0.00 | 4.00 |
| Crop cost | R$ | 41 | 1,441,461 | 1,733,481 | 24,064.18 | 9,197,896.00 |
| Fixed cost | R$ | 41 | 124,003 | 14,944.66 | 99,421.11 | 133,322.90 |

# appendix c

Table C1 - Climate Change Impact on Sugarcane Production - Maximum Temperature

|  | (1) | (2) | (3) | (4) | (5) | (6) | (7) | (8) | (9) | (10) | (11) |
| --- | --- | --- | --- | --- | --- | --- | --- | --- | --- | --- | --- |
| VARIABLES | Yield  SP801816 | Yield  RB867515 | Yield  IACSP955000 | Yield  IACSP955094 | Yield  RB855156 | Yield  RB966928 | Yield  CTC4 | Yield  IAC911099 | Yield  RB855453 | Yield  RB965902 | Yield  CTC9003 |
|  |  |  |  |  |  |  |  |  |  |  |  |
| Precipitation | 0.00823 | -0.0705 | -0.0418 | -0.0299 | -0.0349* | -0.00838 | -0.0521 | 0.000906 | 0.0846 | -0.0185 | -0.00219 |
|  | (0.0421) | (0.0827) | (0.0412) | (0.0250) | (0.0188) | (0.0934) | (0.0828) | (0.0402) | (0.0502) | (0.0205) | (0.0527) |
| Max Temperature | 6.565 | -2.542 | 0.436 | 0.00115 | 0.673 | 4.552 | -4.918 | 0.231 | 1.409 | 0.657 | 1.394 |
|  | (13.14) | (5.010) | (1.668) | (0.795) | (0.489) | (3.198) | (5.532) | (0.899) | (1.636) | (0.599) | (2.203) |
| CO_2_ Emission | 1.74e-06 | 1.64e-05 | -1.29e-06 | 1.26e-06 | 2.56e-06 | 1.66e-05 | -3.07e-06 | -5.75e-07 | 9.78e-07 | -4.46e-07 | 5.73e-06 |
|  | (1.77e-06) | (1.03e-05) | (6.73e-06) | (3.99e-06) | (1.78e-06) | (1.15e-05) | (1.13e-05) | (4.18e-06) | (5.65e-06) | (2.20e-06) | (1.10e-05) |
| CutSP801816 | 14.74 |  |  |  |  |  |  |  |  |  |  |
|  | (10.09) |  |  |  |  |  |  |  |  |  |  |
| CutRB867515 |  | 13.57*** |  |  |  |  |  |  |  |  |  |
|  |  | (2.135) |  |  |  |  |  |  |  |  |  |
| CutIACSP955000 |  |  | 13.97*** |  |  |  |  |  |  |  |  |
|  |  |  | (2.106) |  |  |  |  |  |  |  |  |
| CutIACSP955094 |  |  |  | 27.71*** |  |  |  |  |  |  |  |
|  |  |  |  | (6.432) |  |  |  |  |  |  |  |
| CutRB855156 |  |  |  |  | 26.04*** |  |  |  |  |  |  |
|  |  |  |  |  | (2.904) |  |  |  |  |  |  |
| CutRB966928 |  |  |  |  |  | 34.00*** |  |  |  |  |  |
|  |  |  |  |  |  | (8.213) |  |  |  |  |  |
| CutCTC4 |  |  |  |  |  |  | 15.08*** |  |  |  |  |
|  |  |  |  |  |  |  | (3.213) |  |  |  |  |
| CutIAC911099 |  |  |  |  |  |  |  | 22.57*** |  |  |  |
|  |  |  |  |  |  |  |  | (2.671) |  |  |  |
| CutRB855453 |  |  |  |  |  |  |  |  | 13.73*** |  |  |
|  |  |  |  |  |  |  |  |  | (1.866) |  |  |
| CutRB965902 |  |  |  |  |  |  |  |  |  | 27.82*** |  |
|  |  |  |  |  |  |  |  |  |  | (6.418) |  |
| CutCTC9003 |  |  |  |  |  |  |  |  |  |  | 29.10*** |
|  |  |  |  |  |  |  |  |  |  |  | (5.741) |
| Crop Cost | 5.24e-06 | -4.73e-06 | 9.06e-07 | -2.45e-07 | -2.64e-06 | -1.65e-06 | 4.65e-06 | -7.82e-08 | 6.52e-06 | -8.66e-06* | -3.49e-07 |
|  | (5.55e-06) | (3.27e-06) | (2.34e-06) | (4.29e-07) | (1.98e-06) | (6.58e-06) | (3.66e-06) | (2.22e-06) | (5.26e-06) | (4.67e-06) | (1.91e-06) |
| Fixed Cost | -8.54e-05 | -0.000315 | 0.000110 | -0.000280 | 0.000156 | 0.000467 | -0.000218 | 6.82e-05 | -0.000221 | 0.000295* | -1.83e-05 |
|  | (0.000230) | (0.000383) | (0.000199) | (0.000198) | (0.000106) | (0.000531) | (0.000427) | (0.000108) | (0.000355) | (0.000156) | (0.000221) |
| Constant | -269.8 | 233.3 | 31.06 | 75.27 | 0.695 | -240.9 | 310.7 | -16.45 | -140.9 | -28.13 | -54.63 |
|  | (501.4) | (257.7) | (124.3) | (69.69) | (21.92) | (300.7) | (275.8) | (84.00) | (99.80) | (44.12) | (160.4) |
|  |  |  |  |  |  |  |  |  |  |  |  |
| Observations | 41 | 41 | 41 | 41 | 41 | 41 | 41 | 41 | 41 | 41 | 41 |
| R-squared | 0.646 | 0.600 | 0.781 | 0.762 | 0.919 | 0.578 | 0.557 | 0.874 | 0.676 | 0.870 | 0.756 |

Robust standard errors in parentheses

*** p<0.01, ** p<0.05, * p<0.1

Table C2 - Climate Change Impact on Sugarcane Production - Minimum Temperature

|  | (1) | (2) | (3) | (4) | (5) | (6) | (7) | (8) | (9) | (10) | (11) |
| --- | --- | --- | --- | --- | --- | --- | --- | --- | --- | --- | --- |
| VARIABLES | Yield  SP801816 | Yield  RB867515 | Yield  IACSP955000 | Yield  IACSP955094 | Yield  RB855156 | Yield  RB966928 | Yield  CTC4 | Yield  IAC911099 | Yield  RB855453 | Yield  RB965902 | Yield  CTC9003 |
|  |  |  |  |  |  |  |  |  |  |  |  |
| Precipitation | 0.729 | -0.350 | 0.00617 | -0.0298 | 0.0389 | 0.492 | -0.592 | 0.0262 | 0.239 | 0.0537 | 0.151 |
|  | (1.411) | (0.566) | (0.202) | (0.106) | (0.0448) | (0.427) | (0.624) | (0.129) | (0.184) | (0.0730) | (0.291) |
| Minimum Temperature | 41.68 | -16.14 | 2.771 | 0.00731 | 4.272 | 28.90 | -31.23 | 1.464 | 8.945 | 4.174 | 8.849 |
|  | (83.41) | (31.80) | (10.59) | (5.050) | (3.108) | (20.31) | (35.12) | (5.705) | (10.39) | (3.800) | (13.98) |
| CO_2_ Emission | 1.74e-06 | 1.64e-05 | -1.29e-06 | 1.26e-06 | 2.56e-06 | 1.66e-05 | -3.07e-06 | -5.75e-07 | 9.78e-07 | -4.46e-07 | 5.73e-06 |
|  | (1.77e-06) | (1.03e-05) | (6.73e-06) | (3.99e-06) | (1.78e-06) | (1.15e-05) | (1.13e-05) | (4.18e-06) | (5.65e-06) | (2.20e-06) | (1.10e-05) |
| CutSP801816 | 14.74 |  |  |  |  |  |  |  |  |  |  |
|  | (10.09) |  |  |  |  |  |  |  |  |  |  |
| CutRB867515 |  | 13.57*** |  |  |  |  |  |  |  |  |  |
|  |  | (2.135) |  |  |  |  |  |  |  |  |  |
| CutIACSP955000 |  |  | 13.97*** |  |  |  |  |  |  |  |  |
|  |  |  | (2.106) |  |  |  |  |  |  |  |  |
| CutIACSP955094 |  |  |  | 27.71*** |  |  |  |  |  |  |  |
|  |  |  |  | (6.432) |  |  |  |  |  |  |  |
| CutRB855156 |  |  |  |  | 26.04*** |  |  |  |  |  |  |
|  |  |  |  |  | (2.904) |  |  |  |  |  |  |
| CutRB966928 |  |  |  |  |  | 34.00*** |  |  |  |  |  |
|  |  |  |  |  |  | (8.213) |  |  |  |  |  |
| CutCTC4 |  |  |  |  |  |  | 15.08*** |  |  |  |  |
|  |  |  |  |  |  |  | (3.213) |  |  |  |  |
| CutIAC911099 |  |  |  |  |  |  |  | 22.57*** |  |  |  |
|  |  |  |  |  |  |  |  | (2.671) |  |  |  |
| CutRB855453 |  |  |  |  |  |  |  |  | 13.73*** |  |  |
|  |  |  |  |  |  |  |  |  | (1.866) |  |  |
| CutRB965902 |  |  |  |  |  |  |  |  |  | 27.82*** |  |
|  |  |  |  |  |  |  |  |  |  | (6.418) |  |
| CutCTC9003 |  |  |  |  |  |  |  |  |  |  | 29.10*** |
|  |  |  |  |  |  |  |  |  |  |  | (5.741) |
| Crop Cost | 5.24e-06 | -4.73e-06 | 9.06e-07 | -2.45e-07 | -2.64e-06 | -1.65e-06 | 4.65e-06 | -7.82e-08 | 6.52e-06 | -8.66e-06* | -3.49e-07 |
|  | (5.55e-06) | (3.27e-06) | (2.34e-06) | (4.29e-07) | (1.98e-06) | (6.58e-06) | (3.66e-06) | (2.22e-06) | (5.26e-06) | (4.67e-06) | (1.91e-06) |
| Fixed Cost | -8.54e-05 | -0.000315 | 0.000110 | -0.000280 | 0.000156 | 0.000467 | -0.000218 | 6.82e-05 | -0.000221 | 0.000295* | -1.83e-05 |
|  | (0.000230) | (0.000383) | (0.000199) | (0.000198) | (0.000106) | (0.000531) | (0.000427) | (0.000108) | (0.000355) | (0.000156) | (0.000221) |
| Constant | -1,304 | 633.9 | -37.71 | 75.08 | -105.3 | -958.2 | 1,086 | -52.79 | -362.9 | -131.7 | -274.2 |
|  | (2,570) | (1,023) | (380.7) | (183.3) | (93.28) | (796.8) | (1,126) | (221.8) | (348.9) | (135.8) | (503.8) |
|  |  |  |  |  |  |  |  |  |  |  |  |
| Observations | 41 | 41 | 41 | 41 | 41 | 41 | 41 | 41 | 41 | 41 | 41 |
| R-squared | 0.646 | 0.600 | 0.781 | 0.762 | 0.919 | 0.578 | 0.557 | 0.874 | 0.676 | 0.870 | 0.756 |

Robust standard errors in parentheses

*** p<0.01, ** p<0.05, * p<0.1

# appendix d

Table D1 – Bootstrap Regression Results – Mean Temperature

|  | (1) | (2) | (3) | (4) | (5) | (6) | (7) | (8) | (9) | (10) | (11) |
| --- | --- | --- | --- | --- | --- | --- | --- | --- | --- | --- | --- |
| VARIABLES | Yield  SP801816 | Yield  RB867515 | Yield  IACSP955000 | Yield  IACSP955094 | Yield  RB855156 | Yield  RB966928 | Yield  CTC4 | Yield  IAC911099 | Yield  RB855453 | Yield  RB965902 | Yield  CTC9003 |
|  |  |  |  |  |  |  |  |  |  |  |  |
| Precipitation | 0.106 | -0.109** | -0.0352 | -0.0299 | -0.0249 | 0.0596 | -0.126 | 0.00435 | 0.106* | -0.00870 | 0.0186 |
|  | (0.486) | (0.0506) | (0.0638) | (0.0469) | (0.0188) | (0.141) | (0.0913) | (0.0539) | (0.0606) | (0.0281) | (0.241) |
| Mean Temperature | 11.34 | -4.393 | 0.754 | 0.00199 | 1.163 | 7.866 | -8.498 | 0.398 | 2.434 | 1.136 | 2.408 |
|  | (71.18) | (2.999) | (3.181) | (1.782) | (0.931) | (5.795) | (5.357) | (1.787) | (3.096) | (1.142) | (9.321) |
| CO_2_ Emission | 1.74e-06 | 1.64e-05*** | -1.29e-06 | 1.26e-06 | 2.56e-06 | 1.66e-05 | -3.07e-06 | -5.75e-07 | 9.78e-07 | -4.46e-07 | 5.73e-06 |
|  | (2.19e-06) | (6.25e-06) | (7.66e-06) | (5.18e-06) | (2.00e-06) | (1.31e-05) | (9.57e-06) | (4.96e-06) | (6.66e-06) | (2.55e-06) | (2.82e-05) |
| CutSP801816 | 14.74 |  |  |  |  |  |  |  |  |  |  |
|  | (33.07) |  |  |  |  |  |  |  |  |  |  |
| CutRB867515 |  | 13.57*** |  |  |  |  |  |  |  |  |  |
|  |  | (2.103) |  |  |  |  |  |  |  |  |  |
| CutIACSP955000 |  |  | 13.97*** |  |  |  |  |  |  |  |  |
|  |  |  | (2.364) |  |  |  |  |  |  |  |  |
| CutIACSP955094 |  |  |  | 27.71** |  |  |  |  |  |  |  |
|  |  |  |  | (10.89) |  |  |  |  |  |  |  |
| CutRB855156 |  |  |  |  | 26.04*** |  |  |  |  |  |  |
|  |  |  |  |  | (3.103) |  |  |  |  |  |  |
| CutRB966928 |  |  |  |  |  | 34.00*** |  |  |  |  |  |
|  |  |  |  |  |  | (8.611) |  |  |  |  |  |
| CutCTC4 |  |  |  |  |  |  | 15.08*** |  |  |  |  |
|  |  |  |  |  |  |  | (4.960) |  |  |  |  |
| CutIAC911099 |  |  |  |  |  |  |  | 22.57*** |  |  |  |
|  |  |  |  |  |  |  |  | (2.814) |  |  |  |
| CutRB855453 |  |  |  |  |  |  |  |  | 13.73*** |  |  |
|  |  |  |  |  |  |  |  |  | (2.176) |  |  |
| CutRB965902 |  |  |  |  |  |  |  |  |  | 27.82*** |  |
|  |  |  |  |  |  |  |  |  |  | (6.853) |  |
| CutCTC9003 |  |  |  |  |  |  |  |  |  |  | 29.10** |
|  |  |  |  |  |  |  |  |  |  |  | (14.22) |
| Crop Cost | 5.24e-06 | -4.73e-06 | 9.06e-07 | -2.45e-07 | -2.64e-06 | -1.65e-06 | 4.65e-06 | -7.82e-08 | 6.52e-06 | -8.66e-06* | -3.49e-07 |
|  | (6.49e-06) | (3.83e-06) | (3.83e-06) | (9.47e-07) | (2.84e-06) | (8.94e-06) | (5.34e-06) | (3.65e-06) | (6.76e-06) | (5.09e-06) | (3.36e-06) |
| Fixed Cost | -8.54e-05 | -0.000315 | 0.000110 | -0.000280 | 0.000156 | 0.000467 | -0.000218 | 6.82e-05 | -0.000221 | 0.000295* | -1.83e-05 |
|  | (0.000229) | (0.000435) | (0.000230) | (0.000206) | (0.000125) | (0.000577) | (0.000443) | (0.000142) | (0.000385) | (0.000168) | (0.000223) |
| Constant | -410.5 | 287.8** | 21.70 | 75.24 | -13.73 | -338.5 | 416.2 | -21.39 | -171.1 | -42.22 | -84.51 |
|  | (2,372) | (140.7) | (176.4) | (113.9) | (38.46) | (378.8) | (256.2) | (114.5) | (152.1) | (63.75) | (581.0) |
|  |  |  |  |  |  |  |  |  |  |  |  |
| Observations | 41 | 41 | 41 | 41 | 41 | 41 | 41 | 41 | 41 | 41 | 41 |
| R-squared | 0.646 | 0.600 | 0.781 | 0.762 | 0.919 | 0.578 | 0.557 | 0.874 | 0.676 | 0.870 | 0.756 |

Standard Errors in parentheses

*** p<0.01, ** p<0.05, * p<0.1

Table D2 - Bootstrap Regression Results – Maximum Temperature

|  | (1) | (2) | (3) | (4) | (5) | (6) | (7) | (8) | (9) | (10) | (11) |
| --- | --- | --- | --- | --- | --- | --- | --- | --- | --- | --- | --- |
| VARIABLES | YieldSP801816 | YieldRB867515 | YieldIACSP955000 | YieldIACSP955094 | YieldRB855156 | YieldRB966928 | YieldCTC4 | YieldIAC911099 | YieldRB855453 | YieldRB965902 | YieldCTC9003 |
|  |  |  |  |  |  |  |  |  |  |  |  |
| Precipitation | 0.00823 | -0.0705 | -0.0418 | -0.0299 | -0.0349* | -0.00838 | -0.0521 | 0.000906 | 0.0846 | -0.0185 | -0.00219 |
|  | (0.133) | (0.0480) | (0.0461) | (0.0334) | (0.0204) | (0.0986) | (0.0657) | (0.0408) | (0.0530) | (0.0237) | (0.169) |
| Max Temperature | 6.565 | -2.542 | 0.436 | 0.00115 | 0.673 | 4.552 | -4.918 | 0.231 | 1.409 | 0.657 | 1.394 |
|  | (41.07) | (1.711) | (1.838) | (1.007) | (0.542) | (3.322) | (3.126) | (0.978) | (1.813) | (0.664) | (5.636) |
| CO_2_ Emission | 1.74e-06 | 1.64e-05*** | -1.29e-06 | 1.26e-06 | 2.56e-06 | 1.66e-05 | -3.07e-06 | -5.75e-07 | 9.78e-07 | -4.46e-07 | 5.73e-06 |
|  | (2.15e-06) | (6.27e-06) | (7.69e-06) | (5.01e-06) | (2.02e-06) | (1.32e-05) | (9.57e-06) | (4.68e-06) | (6.67e-06) | (2.59e-06) | (2.95e-05) |
| CutSP801816 | 14.74 |  |  |  |  |  |  |  |  |  |  |
|  | (32.97) |  |  |  |  |  |  |  |  |  |  |
| CutRB867515 |  | 13.57*** |  |  |  |  |  |  |  |  |  |
|  |  | (2.204) |  |  |  |  |  |  |  |  |  |
| CutIACSP955000 |  |  | 13.97*** |  |  |  |  |  |  |  |  |
|  |  |  | (2.398) |  |  |  |  |  |  |  |  |
| CutIACSP955094 |  |  |  | 27.71** |  |  |  |  |  |  |  |
|  |  |  |  | (10.95) |  |  |  |  |  |  |  |
| CutRB855156 |  |  |  |  | 26.04*** |  |  |  |  |  |  |
|  |  |  |  |  | (3.071) |  |  |  |  |  |  |
| CutRB966928 |  |  |  |  |  | 34.00*** |  |  |  |  |  |
|  |  |  |  |  |  | (8.624) |  |  |  |  |  |
| CutCTC4 |  |  |  |  |  |  | 15.08*** |  |  |  |  |
|  |  |  |  |  |  |  | (5.083) |  |  |  |  |
| CutIAC911099 |  |  |  |  |  |  |  | 22.57*** |  |  |  |
|  |  |  |  |  |  |  |  | (2.815) |  |  |  |
| CutRB855453 |  |  |  |  |  |  |  |  | 13.73*** |  |  |
|  |  |  |  |  |  |  |  |  | (2.166) |  |  |
| CutRB965902 |  |  |  |  |  |  |  |  |  | 27.82*** |  |
|  |  |  |  |  |  |  |  |  |  | (6.780) |  |
| CutCTC9003 |  |  |  |  |  |  |  |  |  |  | 29.10** |
|  |  |  |  |  |  |  |  |  |  |  | (14.76) |
| Crop Cost | 5.24e-06 | -4.73e-06 | 9.06e-07 | -2.45e-07 | -2.64e-06 | -1.65e-06 | 4.65e-06 | -7.82e-08 | 6.52e-06 | -8.66e-06* | -3.49e-07 |
|  | (6.44e-06) | (3.84e-06) | (3.83e-06) | (1.03e-06) | (2.82e-06) | (8.75e-06) | (5.30e-06) | (3.59e-06) | (6.89e-06) | (5.10e-06) | (3.32e-06) |
| Fixed Cost | -8.54e-05 | -0.000315 | 0.000110 | -0.000280 | 0.000156 | 0.000467 | -0.000218 | 6.82e-05 | -0.000221 | 0.000295* | -1.83e-05 |
|  | (0.000227) | (0.000431) | (0.000229) | (0.000203) | (0.000126) | (0.000568) | (0.000448) | (0.000138) | (0.000390) | (0.000170) | (0.000228) |
| Constant | -269.8 | 233.3** | 31.06 | 75.27 | 0.695 | -240.9 | 310.7 | -16.45 | -140.9 | -28.13 | -54.63 |
|  | (1,484) | (113.7) | (138.3) | (92.63) | (29.18) | (307.6) | (196.6) | (88.59) | (118.1) | (50.47) | (487.3) |
|  |  |  |  |  |  |  |  |  |  |  |  |
| Observations | 41 | 41 | 41 | 41 | 41 | 41 | 41 | 41 | 41 | 41 | 41 |
| R-squared | 0.646 | 0.600 | 0.781 | 0.762 | 0.919 | 0.578 | 0.557 | 0.874 | 0.676 | 0.870 | 0.756 |

Standard errors in parentheses

*** p<0.01, ** p<0.05, * p<0.1

Table D3 - Bootstrap Regression Results – Minimum Temperature

|  | (1) | (2) | (3) | (4) | (5) | (6) | (7) | (8) | (9) | (10) | (11) |
| --- | --- | --- | --- | --- | --- | --- | --- | --- | --- | --- | --- |
| VARIABLES | YieldSP801816 | YieldRB867515 | YieldIACSP955000 | YieldIACSP955094 | YieldRB855156 | YieldRB966928 | YieldCTC4 | YieldIAC911099 | YieldRB855453 | YieldRB965902 | YieldCTC9003 |
|  |  |  |  |  |  |  |  |  |  |  |  |
| Precipitation | 0.729 | -0.350* | 0.00617 | -0.0298 | 0.0389 | 0.492 | -0.592 | 0.0262 | 0.239 | 0.0537 | 0.151 |
|  | (4.377) | (0.191) | (0.221) | (0.145) | (0.0524) | (0.440) | (0.365) | (0.144) | (0.208) | (0.0814) | (0.740) |
| Minimum Temperature | 41.68 | -16.14 | 2.771 | 0.00731 | 4.272 | 28.90 | -31.23 | 1.464 | 8.945 | 4.174 | 8.849 |
|  | (260.5) | (10.98) | (11.39) | (6.702) | (3.366) | (20.88) | (19.64) | (6.503) | (11.45) | (4.164) | (33.73) |
| CO_2_ Emission | 1.74e-06 | 1.64e-05*** | -1.29e-06 | 1.26e-06 | 2.56e-06 | 1.66e-05 | -3.07e-06 | -5.75e-07 | 9.78e-07 | -4.46e-07 | 5.73e-06 |
|  | (2.18e-06) | (6.25e-06) | (7.46e-06) | (5.33e-06) | (1.97e-06) | (1.29e-05) | (9.43e-06) | (4.94e-06) | (6.69e-06) | (2.60e-06) | (2.78e-05) |
| CutSP801816 | 14.74 |  |  |  |  |  |  |  |  |  |  |
|  | (32.94) |  |  |  |  |  |  |  |  |  |  |
| CutRB867515 |  | 13.57*** |  |  |  |  |  |  |  |  |  |
|  |  | (2.214) |  |  |  |  |  |  |  |  |  |
| CutIACSP955000 |  |  | 13.97*** |  |  |  |  |  |  |  |  |
|  |  |  | (2.388) |  |  |  |  |  |  |  |  |
| CutIACSP955094 |  |  |  | 27.71** |  |  |  |  |  |  |  |
|  |  |  |  | (11.20) |  |  |  |  |  |  |  |
| CutRB855156 |  |  |  |  | 26.04*** |  |  |  |  |  |  |
|  |  |  |  |  | (2.995) |  |  |  |  |  |  |
| CutRB966928 |  |  |  |  |  | 34.00*** |  |  |  |  |  |
|  |  |  |  |  |  | (8.522) |  |  |  |  |  |
| CutCTC4 |  |  |  |  |  |  | 15.08*** |  |  |  |  |
|  |  |  |  |  |  |  | (5.011) |  |  |  |  |
| CutIAC911099 |  |  |  |  |  |  |  | 22.57*** |  |  |  |
|  |  |  |  |  |  |  |  | (2.824) |  |  |  |
| CutRB855453 |  |  |  |  |  |  |  |  | 13.73*** |  |  |
|  |  |  |  |  |  |  |  |  | (2.187) |  |  |
| CutRB965902 |  |  |  |  |  |  |  |  |  | 27.82*** |  |
|  |  |  |  |  |  |  |  |  |  | (6.828) |  |
| CutCTC9003 |  |  |  |  |  |  |  |  |  |  | 29.10** |
|  |  |  |  |  |  |  |  |  |  |  | (14.54) |
| Crop Cost | 5.24e-06 | -4.73e-06 | 9.06e-07 | -2.45e-07 | -2.64e-06 | -1.65e-06 | 4.65e-06 | -7.82e-08 | 6.52e-06 | -8.66e-06* | -3.49e-07 |
|  | (6.53e-06) | (3.90e-06) | (3.79e-06) | (9.73e-07) | (2.83e-06) | (8.80e-06) | (5.46e-06) | (3.57e-06) | (6.87e-06) | (5.11e-06) | (3.39e-06) |
| Fixed Cost | -8.54e-05 | -0.000315 | 0.000110 | -0.000280 | 0.000156 | 0.000467 | -0.000218 | 6.82e-05 | -0.000221 | 0.000295* | -1.83e-05 |
|  | (0.000233) | (0.000435) | (0.000250) | (0.000204) | (0.000125) | (0.000565) | (0.000454) | (0.000138) | (0.000385) | (0.000170) | (0.000225) |
| Constant | -1,304 | 633.9* | -37.71 | 75.08 | -105.3 | -958.2 | 1,086 | -52.79 | -362.9 | -131.7 | -274.2 |
|  | (7,948) | (356.9) | (412.2) | (254.1) | (105.7) | (816.4) | (668.0) | (250.6) | (392.6) | (150.2) | (1,294) |
|  |  |  |  |  |  |  |  |  |  |  |  |
| Observations | 41 | 41 | 41 | 41 | 41 | 41 | 41 | 41 | 41 | 41 | 41 |
| R-squared | 0.646 | 0.600 | 0.781 | 0.762 | 0.919 | 0.578 | 0.557 | 0.874 | 0.676 | 0.870 | 0.756 |

Standard errors in parentheses

*** p<0.01, ** p<0.05, * p<0.1
